# A Metagenomic Nanopore Sequence Analysis Combined with Conventional Screening and Spectroscopic Methods for Deciphering the Antimicrobial Metabolites Produced by *Alcaligenes faecalis* Soil Isolate MZ921504

**DOI:** 10.3390/antibiotics10111382

**Published:** 2021-11-11

**Authors:** Mohamed A. Eltokhy, Bishoy T. Saad, Wafaa N. Eltayeb, Mona R. El-Ansary, Khaled M. Aboshanab, Mohamed S. E. Ashour

**Affiliations:** 1 Department of Microbiology, Faculty of Pharmacy, Misr International University (MIU), Cairo 19648, Egypt; mohammad.ashraf@miuegypt.edu.eg (M.A.E.); wafaa.eltayeb@miuegypt.edu.eg (W.N.E.); 2 Department of Bioinformatics, HITS Solutions Co., Cairo 11765, Egypt; bishoyth@hitssolutions.com; 3 Department of Biochemistry, Modern University for Technology and Information (MTI), Cairo 12055, Egypt; Mona.El-Ansary@pharm.mti.edu.eg; 4 Department of Microbiology and Immunology, Faculty of Pharmacy, Ain Shams University, Organization of African Unity St., Cairo 11566, Egypt; 5 Department of Microbiology and Immunology, Faculty of Pharmacy, Al-Azhar University, Cairo 11651, Egypt; seifashour@hotmail.com

**Keywords:** *Alcaligenes faecalis*, LC/MS, metagenomics, ectoine, bacillibactin, quinolobactin, burkholderic acid

## Abstract

The continuous development of multidrug resistance pathogens with limited therapeutic options has become a great problem globally that impose sever health hazards. Accordingly, searching for of new antimicrobials became an urgent demand and great challenge. Soil significantly have been associated with several species that are antibiotic producers. In this study, combination of conventional screening methods with Liquid chromatography- Mass spectroscopy (LC/MS) and metagenomic nanopore sequence analysis have been conducted for the deciphering the active metabolites produced by soil isolate(s). Preliminary soil screening resulted in a Gram-negative isolate identified via 16S ribosomal RNA as *Alcaligenes faecalis* isolate MZ921504 with promising antimicrobial activities against wide range of MDR gram-positive and gram-negative pathogens. The LC/MS analysis of the metabolites of *A. faecalis* isolate MZ921504 confirmed the presence of ectoine, bacillibactin, quinolobactin and burkholderic acid. Metagenomics sequence analysis of the soil sample (NCBI GenBank accession PRJNA771993) revealed the presence of conserved biosynthetic gene clusters of ectoine, bacteriocin, bacillibactin, quinolobactin, terpene and burkholderic acid of *A. faecalis*. In conclusion, *A. faecalis* isolate MZ921504 is a promising source for antimicrobial metabolites. LC/MS spectral analysis and third generation sequencing tools followed by secondary metabolite gene clusters analysis are useful methods to predict the nature of the antimicrobial metabolites.

## 1. Introduction

In 2004, the world health organization (WHO) declared that the search for new medicines to combat multidrug resistant (MDR) is the number one area for research that will impact public health [1]. The extensive spread of bacterial resistance became a public health issue that needs fast intervention [2]. The unsatisfactory result in combating MDR could mean a literal return to pre-antibiotic era for many infections [3]. Countries already started protocols to prevent further spread of resistant bacterial strains, with non-consistent results [4]. The alarming rate of MDR made it obligatory to search for new approaches to combat MDR [5]. The natural compounds that are produced from bacteria and fungi are secondary metabolites. *Actinomycetaceae*, bacterial family, has been associated with most of new and old antimicrobials produced by bacteria. The first 30 years of antibiotic discovery, from 1940s to 1970s, was dependent on phenotypic screening which led to the discovery of streptomycin and other antibiotics [6]. A projected approach is searching for new antimicrobial agents from natural sources [5]. The huge diversity of soil serves a great potential for mining novel antimicrobials from soil microbiota [7].

Although bacteria from the soil has been studied for more than a century, but still most soil bacterial 16S ribosomal RNA (rRNA) sequence do not match what we have in the GenBank [8]. The advancement in sequence technology made it possible to revisit the path of discovery of new antimicrobials naturally, without revisiting old pathways through trial and error [6]. Developed genetic engineering and genetic learnings together with high throughput detection approaches increases the high probability for novel natural product discovery and production [6].

In the second decade in sequencing, metagenomics analysis was introduced. Metagenomics analysis covers all DNA present in a sample [9]. Metagenomics comprises the sampling of various genomic sequences from a specific community. It has been used solely to microbial habitats. It serves as an impartial platform for microbial diversity and metabolic potential of a microbial community. Metagenomics steered the wheel back to discovery of antimicrobials from natural sources after abandoning this way for some time due to the elevated rediscovery rates of antimicrobials [10]. Bioinformatics analysis and systematic characterization make it possible to explore hidden biosynthetic gene clusters. The culture independent approach contribute to the discovery of malacidins an antimicrobial group that have activity against MRSA [10].

The genus *Alcaligenes* is known to have antagonistic activity against other microorganisms [11,12]. *Alcaligenes faecalis* (*A. faecalis*) is an organism isolated from soil or marine environments that exhibit antibacterial activity against other microorganism, thus it serves as a potential organism for antimicrobial extraction and discovery [11,12,13]. *A. faecalis* is Gram negative rod shaped, alkali tolerant and motile [13,14]. It is known to be oxidase and cytochrome oxidase positive, catalase negative, glucose non fermenter, proteolytic and hydrolyze starch [13]. *A. faecalis* was examined for bacteriocin production by examining inhibitory effect against several microorganisms which were *Salmonella*, *Escherichia*, *Serratia*, *Proteus*, *Staphylococci* and Gram-positive aerobic *bacilli* [15]. In a study conducted by Zahir et al. (2013), *A. faecalis* was isolated from soil sample and the extracted antimicrobial showed inhibitory effects against both Gram positive and Gram negative. The antimicrobial agent was extracted using solvent extraction method by ethyl acetate and believed to be a non-proteinaceous substance [16].

Few studies were published on the antimicrobial metabolites produced by *A. faecalis* [17,18,19]. Therefore, the present study aimed to discover the nature of the antimicrobial metabolites from *A. faecalis* isolate recovered from Egyptian soil by using conventional and chromatographic methods and correlating the obtained results with those of nanopore metagenomics biosynthetic gene clusters analysis which is a novel approach for deciphering the antimicrobial metabolites of soil microbiota.

## 2. Results

### 2.1. Screening of the Antimicrobial Activities of the Recovered Bacterial Isolates

Table 1 and Table 2 showed the results of the preliminary screening for the antimicrobial activities of the recovered bacterial isolates. Only eight isolates, coded SS9, SS10, SS11, SS12, SS13, SS14, SS16 and SS17 and six isolates, coded SS9, SS10, SS13, SS14, SS16 and SS17 gave positive results against the tested Gram positive and Gram negative, respectively. None of the recovered isolates gave growth inhibition against the tested *Candida* isolates. Table 1 and Table 2 revealed the inhibition zone of the promising Gram positive *Bacillus* sp. (coded SS10) and Gram-negative isolate (coded SS17). In this study, the Gram-negative isolate (SS17) was selected for further study since few studies were conducted on the Gram-negative strains that showed antimicrobial activities against wide range of MDR Gram positive and Gram-negative pathogens.

### 2.2. Molecular Identification

The16s ribosomal RNA sequencing of the Gram negative isolate coded SS17 revealed 99% identity to NCBI reference sequence of *A. faecalis* strain 32SR17. Therefore, the isolate coded SS17 was identified as *A. faecalis* isolate MZ921504. The RNA sequence was deposited in the GenBank under accession number MZ921504.

### 2.3. The Antimicrobial Activities of the Extracted Metabolite(s) of A. faecalis Isolate MZ921504

Table 3 revealed the results of the antimicrobial activity (measured in zone of inhibition) of the *A. faecalis* isolate MZ921504 of dichloromethane and ethyl acetate solvent extracts.

### 2.4. Characterization of the Antimicrobial Metabolite(s)

#### 2.4.1. TLC Analysis

TLC was carried out for ethyl acetate extracts of *A. faecalis* fermentation product. TLC using solvent system ethyl acetate: methanol (9:1) showed four separated spots. TLC using solvent system ethyl acetate: methylene chloride (9:1) revealed eight separated spots. However, them TLC for solvent system dichloromethane: ethanol (6.5:3.5) showed no significant separation of the spots. Accordingly, the solvent system ethyl acetate: methylene chloride 9:1 was the optimum for better separation of the TLC spots.

#### 2.4.2. LC/MS Analysis

##### Ethyl Acetate Extract of *A. faecalis*

**Figure 1:**
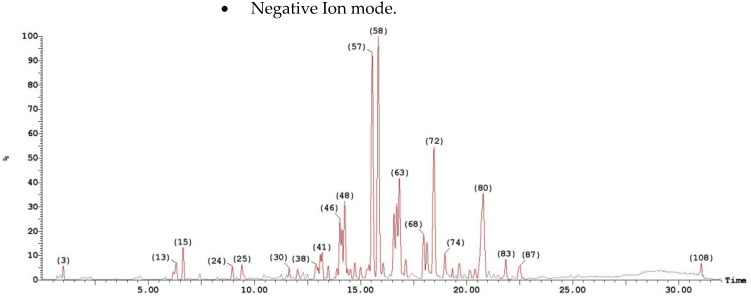

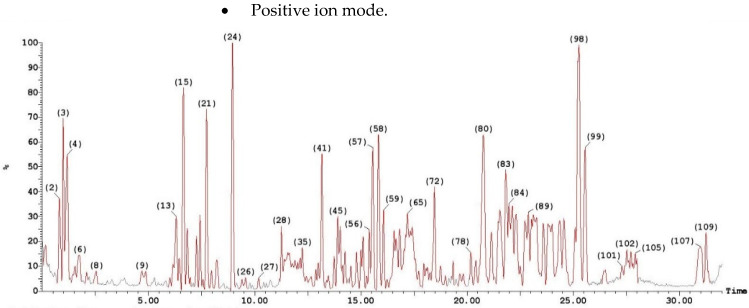
A cell free ethyl acetate extract run on both ESI-MS positive and negative ion mode for antibiotic detection from the isolate *A. faecalis* isolate MZ921504 using XEVO TQD triple quadrupole instrument.

LC/MS was carried on cell free ethyl acetate extract generated 108 peaks in negative ion mode and 109 peaks in positive ion mode illustrated in Figure 1 and each peak had variable masses.

**Figure 2:**
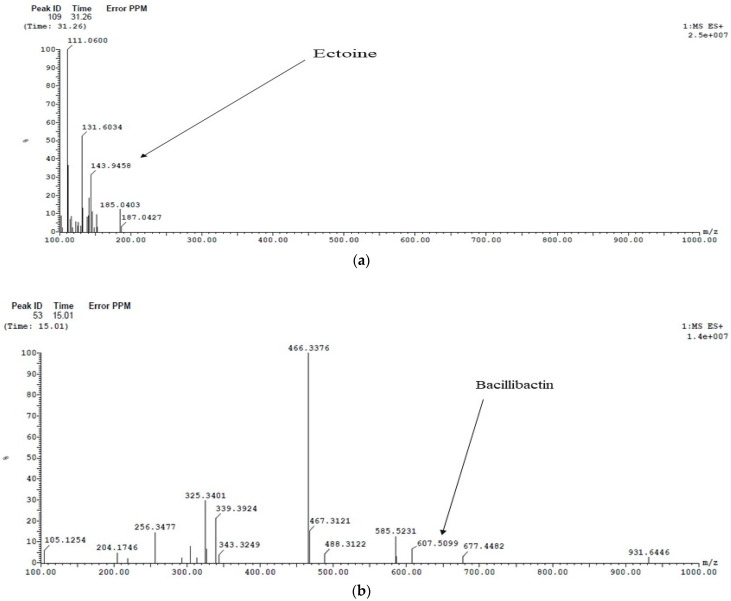

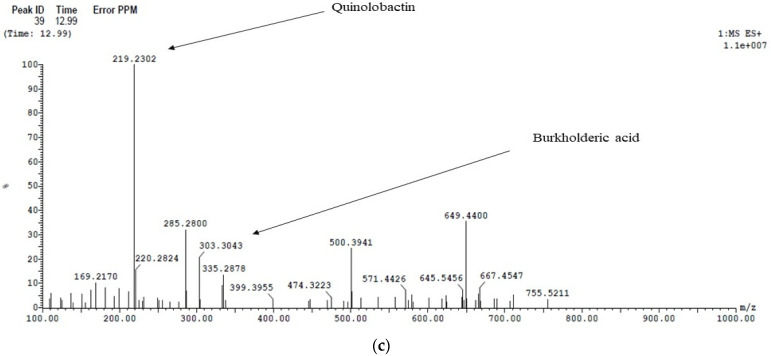
LC/MS analysis of a cell free ethyl acetate extract of *A. faecalis* isolate MZ921504 (**a**) detection of ectoine, (**b**) detection of bacillibactin, (**c**) detection of quinolobactin and Burkholderic acid.

The mass spectra were analyzed for the detection of the secondary metabolite of *A. faecalis* isolate MZ921504 as illustrated in Figure 2. Ectoine was detected at peak 109 at time 31.26 min with *m/z* 143.94. Bacillibactin was detected at peak 53 at time 15.01 min with *m/z* 607.5099. Quinolobactin was detected at peak 39 at time 12.99 min with *m/z* 219.2302 and detection of Burkholderic acid at peak 39 at time 12.99 min with *m/z* 303.3043.

### 2.5. Metagenomics Analysis of the Soil Sample

We observed a range between 500–1080 reads in the sample and control. FastQ quality score per base showed good quality (Phred score range between 10 and <25). There were no duplicate reads. Sequence length (bp) ranged from 250–12,000 bp. Percentage N count (ambiguous) was zero.

Percent abundance of the bacterial phylum present in the soil, shows that the most abundant phylum was *Achromobacter.* The organism with the most abundance is *Achromobacter* sp. MFA1 R4 representing 56% of the sample, followed by *Achromobacter* sp. AONIH1 and *Achromobacter xylosoxidans*, both of 11% abundance. *A. faecalis* belongs to Proteobacteria phylum which presented 0.4% abundance. There are two species related to *Alcaligens* genus present in the soil which are *Alcaligens faecalis* (67%) and *Alcaligens aquatilis* (33%) (Supplementary Material Figure S1). Metagenomics sequences were deposited in the NCBI GenBank under accession number PRJNA771993 (https://www.ncbi.nlm.nih.gov/sra/PRJNA771993, accessed on 7 November 2021).

### 2.6. Identification of Secondary Metabolite(s) Gene Clusters

**Figure 3:**
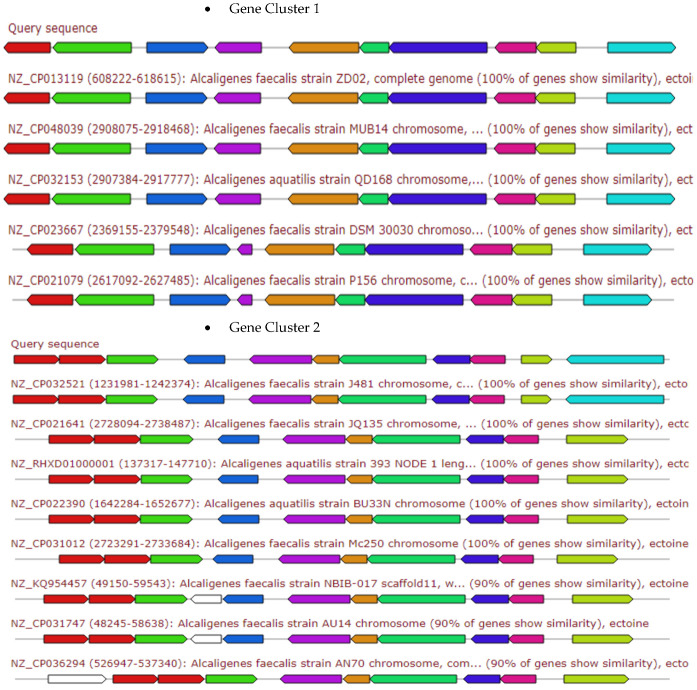
Gene arrangement of top 13 ectoine gene clusters (presented in gene cluster 1 and 2 as compared to the query sequence of *A. faecalis* isolate MZ921504. The putative biosynthetic genes presented in red, additional biosynthetic genes in orange, transport-related genes in blue, regulation-related genes in green color, resistance genes in pink, TTA codon in dark pink.

**Figure 4:**
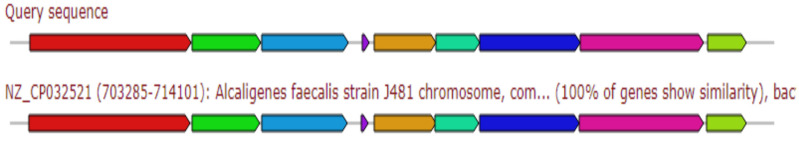
Gene arrangement of bacteriocin gene cluster homologous to query sequence of *A. faecalis* isolate MZ921504. Putative biosynthetic genes presented in red, additional biosynthetic genes in orange, transport-related genes in blue, regulation-related genes in green color, resistance genes in pink, TTA codon in dark pink.

**Figure 5:**
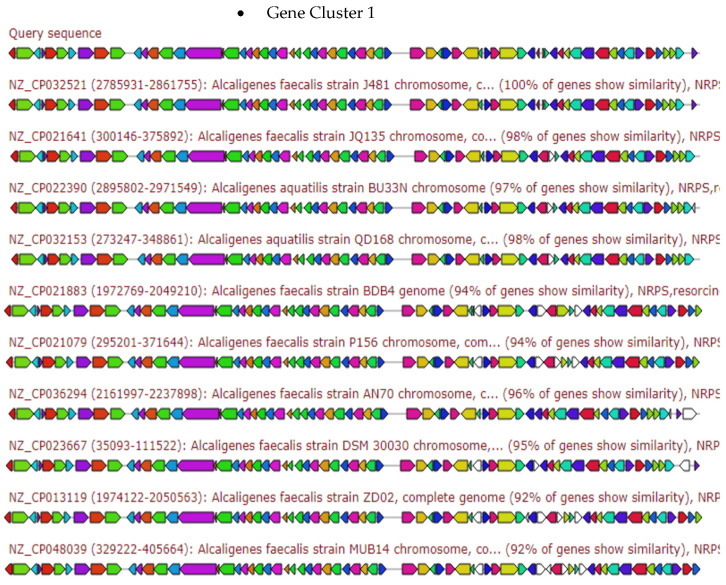

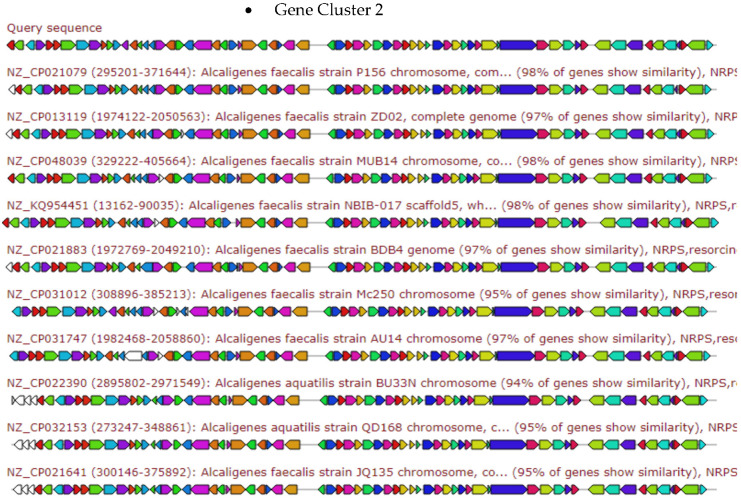
Gene arrangement of resorcinol and multi-modular non-ribosomal peptide synthase (NRPS) gene clusters of various *Alcaligenes* sp. homologous to query sequence of *A. faecalis* isolate MZ921504. Putative biosynthetic genes presented in red, additional biosynthetic genes in orange, transport-related genes in blue, regulation-related genes in green color, resistance genes in pink, TTA codon in dark pink.

**Figure 6:**
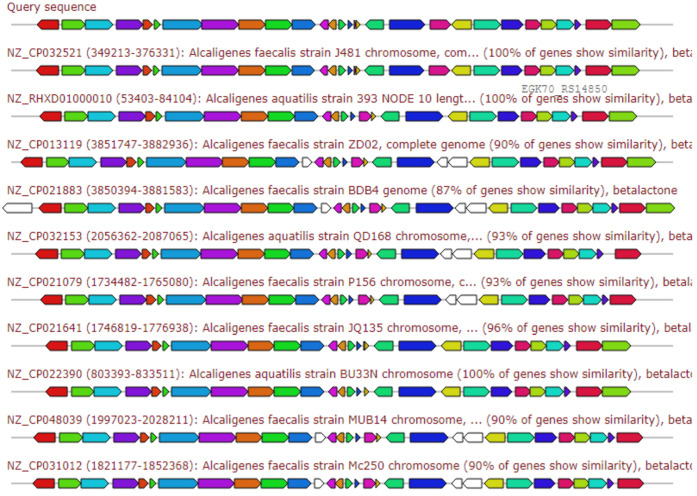
Gene arrangement of β-lactone containing protease inhibitors showing top 10 *Alcaligenes faecalis* strains homologous to query sequence of *A. faecalis* isolate MZ921504. Putative biosynthetic genes presented in red, additional biosynthetic genes in orange, transport-related genes in blue, regulation-related genes in green color, resistance genes in pink, TTA codon in dark pink.

**Figure 7:**
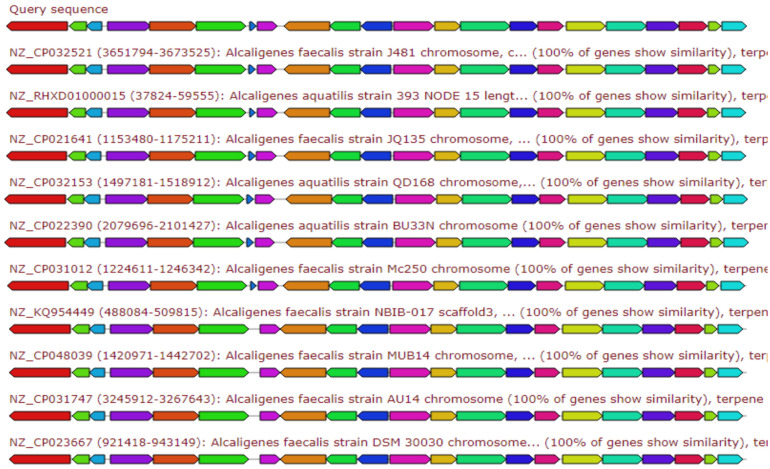
Gene arrangement of the top 10 *Alcaligenes* sp. showing homologous to query sequence of *A. faecalis* isolate MZ921504 for Terpene biosynthetic gene cluster. Putative biosynthetic genes presented in red, additional biosynthetic genes in orange, transport-related genes in blue, regulation-related genes in green color, resistance genes in pink, TTA codon in dark pink.

The Figure 3, Figure 4, Figure 5, Figure 6 and Figure 7 elaborates the similarity between the query genes in *A. faecalis* isolate MZ921504 against the database for the prediction of the produced metabolites, ectoine, bacteriocin, bacillibactin, β-lactones and terpenes, respectively. Genome similarity plays an important role in the synthesis of related compounds to pre-identified genes [20].

#### 2.6.1. Ectoine

Ectoine is a protective substance for bacteria against osmotic pressure (Figure 3). The ectoine biosynthetic gene cluster of *A. faecalis* isolate MZ921504 (presented by the red color) showed homology to the top 13 ectoine gene clusters as depicted in Figure 3.

#### 2.6.2. Bacteriocin or Other Unspecified Linear Ribosomal-Synthesized and Post-Translationally Modified Peptides (RiPP)

Bacteriocins are proteinaceous substance produced by bacteria to inhibit bacterial growth of other bacteria. The bacteriocin biosynthetic gene cluster sequence of *A. faecalis* isolate MZ921504 (query) has a 50% similarity to homologous gene cluster producing methanobactin (Figure 4).

#### 2.6.3. Hybrid Region: Resorcinol and Traditional (Multi-) Modular Non-Ribosomal Peptide Synthase (NRPS)

The gene cluster sequence of *A. faecalis* isolate MZ921504 has a 60% similarity to gene cluster of various *Alcaligenes* sp. producing Bacillibactin (Figure 5).

#### 2.6.4. Beta-Lactone Containing Protease Inhibitor

β-lactones are natural products with potent antifungal and antibacterial activities [21]. The gene cluster sequence of *A. faecalis* isolate MZ921504 has a 26% similarity to gene clusters of various *Alcaligenes* producing quinolobactin as depicted in Figure 6.

#### 2.6.5. Terpene

Terpene is a class of natural products consisting of compounds with the formula (C5H8)n. The gene cluster sequence of *A. faecalis* isolate MZ921504 (query sequence) has a 13% similarity to homologous gene clusters of various *Alcaligenes* producing Burkholderic acid (Figure 7).

## 3. Discussion

The development of new antimicrobial agents has become an urgent demand, as history dictates and proven that natural resources still provide humanity with solutions for development of several natural products including antimicrobials [22]. Soil significantly have been associated with several microorganisms that are antimicrobial producers and have been used extensively for antibiotic production, such as streptomycin from Streptomyces species [22]. *A. faecalis* has not always been associated with antimicrobial production, scarce studies on antimicrobial activity of *A. faecalis* have been published in the last few years [17,18,19]. In this study, it shows a promising approach for new antibiotic discovery as per secondary metabolite gene cluster results [17] and correlating these results with the antimicrobial and LC/MS spectroscopic analysis.

In the present study, preliminary screening was carried out on 15 different soil samples. Of these, one sample revealed the presence *A. faecalis MZ921504* with broad antimicrobial activities against wide range of MDR gram positive and Gram-negative bacteria. This soil sample was selected for further metagenomics analysis of the secondary metabolite biosynthetic gene clusters. The isolated strain of *A. faecalis* from the soil exhibited a broad spectrum of antimicrobial activities. The crude ethyl acetate extract showed activity against colistin resistant *K. pneumoniae*, VRSA, MDR *E. coli* and little activity against *C. albicans*. Other studies showed different inhibitory spectrums. For instance, a strain of *A. faecalis* isolated from Passu glacier in Pakistan produced two antimicrobials which are kalimantacin and tunicamycin [23]. Both showed efficacy against large number of resistant pathogens, such as *S. aureus*, *Bacillus subtilis*, *E. coli*, *K. pneumoniae* and *Pseudomonas aeruginosa* [23]. In accordance to our study, Kapley et al., isolated *A. faecalis* HPC 1271 strain from a waste water treatment system called common effluent treatment plant (CETP) [17]. It showed promising inhibitory effects against MDR bacteria which were *Serratia* sp., *Enterobacter* sp. and reference organisms including, *E. coli*, *Bacillus subtilis* and *Shigella flexneri*, but showed no inhibition against *Salmonella paratyphi* and *S. aureus* [17]. Another strain isolated from Moroccan tannery waste assigned as *A. faecalis* BW1, its ethyl acetate extract showed antimycobacterial activity against *Mycobacterium smegmatiss* [19]. An isolated strain of *A. faecalis* from crude oil field in Malaysia showed inhibitory effect against *Desulfovibrio* sp., a sulfate reducing bacterium, using the isolate chloroform extract [24]. An antipseudomonal activity was exhibited by another strain called *A. faecalis* strain KEM24 isolated from Olabisi Onabanjo University Farmland [18]. The previous studies showed that *A. faecalis* is a promising bacterium as a source for production of antimicrobial compounds.

It was reported that the type of substrate used in the fermentation as a source of carbon, glucose and nitrogen etc. may influence the structure of produced secondary metabolites, such as antimicrobials [25,26]. In a study conducted by Kapley et al., the seed culture was carried out in M9 media [17] and then fermentation was completed in Luria-Bertani (LB) broth at 37 °C, at 150 rpm for 48 h producing antimicrobial [19]. Another study conducted on *A. faecalis* strain producing antimicrobial metabolites against *Desulfovibrio* sp., was cultivated in 1 L of marine broth under rotary agitation condition at 150 rpm at 30 °C for 96 h and metabolites were extracted by chloroform [24]. The different extraction solvents and procedures might have influenced secondary metabolite gene expression and the differences in antimicrobial spectrum. The previous conclusion is clear in our study, the medium used for the bacterial growth and fermentation was starch casein broth and extraction of secondary metabolites was carried out using ethyl acetate followed by dichloromethane.

In the current study the secondary metabolite was characterized using TLC and LC/MS. TLC was used as a preliminary method to determine the difference in Retention Factors (RF) and polarities of the metabolites. LC/MS was used to help identify the inhibitory compounds produced by *A. faecalis*. The LC/MS run reviewed vast diverse compounds found in the cell free extract of ethyl acetate. Correlation of the inhibitory compound was guided by the secondary metabolite gene clusters provided from sequencing and analyzing by antiSMASH. The correlation between the inhibitory compounds and secondary metabolite gene clusters was carried out by matching acquired mass peaks from LC/MS, with highest probability, with pre-identified compounds from secondary metabolite gene cluster analysis. The correlated metabolites in this study were ectoine, bacillibactin, quinolobactin and burkholderic acid. β-lactones such quinolobactin as are natural products that have been described to have potent antifungal and antibacterial activity on human cancer cell lines. Chemically, they are four-membered heterocycles with high ring-strain, electrophilicity and reactivity [21]. Methanobactin gene cluster was also found in gene annotation, but it was not correlated in LC/MS due to the limited detection limit of *m/z* which was 1000 and methanobactin exact mass is 1170.26973 g/mol. Consistent with our study, Kapley et al., characterized of the antimicrobial compound of *A. faecalis* HPC 1271 was carried out by solvent extraction using ethyl acetate followed by metabolite profiling using HPLC and LC-MS [17]. On the other hand, the *A. faecalis* from crude oil field in Malaysia chloroform extract was analyzed using Gas Chromatography Mass Spectrometry (GC-MS). The GC-MS analysis from Malaysian study revealed that the extract contains several antimicrobials, such as decanedioic acid, hexacosane, phenol, 7-hexadecene, 9-eicosene, palmitoleic acid [24].

Metagenomics is known to be a prominent approach for antimicrobial discovery from natural sources [10]. In the current study metagenomics gave an insight of the soil composition collected from Luxor aiming for antibiotic discovery. Although, the most abundant organism is *Achromobacter* sp. MFA1 R4 representing 56%, the organism with the most promising inhibitory effect was *A. faecalis* belonging to the Proteobacteria phylum. Similar to our study, metagenomics analysis of a soil sample in Mars Oasis in the southern maritime Antarctic reported the availability of biosynthetic gene cluster (BGC) in metagenomes [27]. BCGs were found several phyla, such as *Verrucomicrobiota*, *Acidobacteriota* and *Gemmatimonadota* as well as classes of Actinobacteria represented in *gammaproteobacterial*, *Thermoleophilia* and *Acidimicrobiia* [27]. A deep sequencing of Mantag Mangrove forests soil in Malaysia (two different locations) using Illumina HiSeq 2500 platform that the most revealed that the most abundant phyla were Proteobacteria (≅55%), *Firmicutes* (≅11%), *Bacteroidetes* (≅7%), *Chloroflexi*, *Planctomycetes*, *Actinobacteria* and *Cyanobacteria* (≅3–5% each) [28]. Different metagenomics studies represent the different microbial communities with an open realm of opportunities for antimicrobial discovery.

Genome sequencing for secondary metabolite gene clusters was performed on the selected isolate. AntiSMASH analysis showed five gene clusters responsible for production of secondary metabolites. Gene sequencing followed by gene clusters analysis and correlation with LC/MS is a promising technique to guide for the identification of antimicrobial metabolites. The genetic analysis also provided us with information that the isolate produces several metabolites which might be the reason they exhibited a broad spectrum of activity against MDR bacteria and colistin resistant bacteria and VRSA. Gene cluster analysis suggested that *A. faecalis* produces bacteriocins similar compounds with high probability of methanobactin. Another gene cluster is β-lactone containing protease inhibitor with some genetic similarity of qinolobactins where this family of compounds are affiliated with activity against bacteria, fungi, or human cancer cell lines [29]. A 60% gene similarity of bacillibactin is detected within one hybrid region: Resorcinol and traditional (multi-)modular non-ribosomal peptide synthases (NRPS). Bacillibactin is usually produced from *Bacillus* spp. which is an iron regulatory siderophore and exhibits antimicrobial activity [30,31]. A gene cluster also responsible for production of terpenes was found. Several terpene compounds exhibited antimicrobial activity [32,33]. The genetic annotation and assembly conducted in the study by Kapley et al., has several similarities with the current study. Consistent with our study, the genome assembly revealed secondary metabolite gene clusters of ectoine, terpene and NRPS [17]. It also had diverse secondary metabolite gene cluster butyrolactone, phosphonate and type 1 polyketide synthase (T1PKS) [17]. The production of a highly reduced metabolites, such as macrolide antibiotic is correlated with the type 1 modular NRPS which is naturally found in several types of bacteria and fungi as well as symbionts of higher eukaryotes [34,35].

The different inhibitory spectrum might be attributed to several factors apart from the difference in genomic data. The growth conditions that were subjected upon the different isolates might be a contributor to the produced metabolite, that can be the different incubation temperatures and the culture mediums used for the isolate growth [26]. Extraction and analysis methods also contributes to the diversified results in this approach [36]. The microbial community that the bacteria isolated from differs greatly from one environment to another which can influence in gene regulation and expression [27,28].

It is recommended in future work to apply further analytical processing of the extract to completely isolate and identify the antimicrobial metabolites by applying fermentation on a larger scale, to produce sufficient amounts of the metabolites, followed by further purification and fractionation methods.

## 4. Materials and Methods

### 4.1. Isolation and Characterization

The soil samples were collected from different localities in Egypt and were air dried for one week before any further analysis to decrease the microbial count [37]. A suspension of 1 g of each soil sample in 9 mL saline was prepared [38] and was vortexed at 400 rpm for 4 min. [39]. A series of 10-fold serial dilutions were performed from 10-1 to 10-6. For every dilution, about 1 mL was transferred for a surface inoculation on Starch Casein Agar (SCA) [37]. After an incubation period of 7 days, preliminary screening was performed on various bacterial isolates to determine its inhibitory effect against the tested organisms. Microscopical analysis and variable biochemical tests were performed for the preliminary identification of the recovered bacterial isolates [40,41].

### 4.2. Preliminary Screening

In this case, 17 bacterial isolates were recovered from the 15 different soil samples and were screened for the antimicrobial activities against standard *E. coli* ATCC 25922 and three *Staphylococcus (S.) epidermidis* (SE1, SE2, SE3), three vancomycin resistance *S. aureus* (VRSA1, VRSA2, VSRA3), three multidrug resistant (MDR) *K. pneumoniae* (KP1, KP2, KP3), two MDR *E. coli* (EC1, EC2), three *Candida* (*C*.) *albicans* (CA1, CA2, CA3), and three *C. auris* (CS1, CS2, CS3) clinical isolates discharged from the Central Microbiology Lab of Ain Shams Hospital. The antimicrobial resistance patterns of the respective clinical isolates are displayed in Table 4. The 17 isolates were inoculated on Mueller Hinton agar (MHA) and the test organisms were inoculated perpendicularly to the isolate [42,43]. The plates were then incubated for 24 h. at 37 °C. The antimicrobial activities were detected by the observing the inhibition zone around the tested organisms [42,44].

### 4.3. DNA Sequencing of 16S Ribosomal RNA

The 16S ribosomal RNA of the isolate that showed promising and broad antimicrobial activity in the preliminary screening was sequenced and analyzed by GATC Biotech Co., Germany through Sigma Scientific Services Co., Egypt. The provided contig of the 16s ribosomal RNA was aligned and blasted in GenBank database using Basic Local Alignment Search Tool (BLAST, https://blast.ncbi.nlm.nih.gov/Blast.cgi, accessed on 7 November 2021) provided by National Center of Biotechnology Information (NCBI). The results were then expressed as percentage homology between the query sequence and the sequences provided by the database. The alignment of the 16S ribosomal RNA sequence against the sequence from the database was carried out using Multiple Sequence Comparison by Log- Expectation (MUSCLE, https://www.ebi.ac.uk/Tools/msa/muscle/, accessed on 7 November 2021) to retrieve phylogenetic tree. Phylogenetic tree was inferred via likelihood method with a bootstrap analysis (1000 replicates). The 16S ribosomal RNA sequence was deposited in the NCBI GenBank.

### 4.4. Production of the Antimicrobial Metabolite(s) in Shake Flasks

#### Seed Culture Preparation and Production Conditions

The seed culture was prepared by transferring a loopful of fresh culture of the promising bacterial isolate(s) into 50 mL starch casein broth and was incubated at 200 rpm at 35 °C for 24 h. About one ml of the culture was centrifuged for 5 min at 16,000 rpm using a micro centrifuge tube, washed twice with 1 mL sterile saline, and used to inoculate the production flasks (100 mL of casein starch broth × 20 flasks). These flasks were incubated in a shaking incubator (150 rpm) at 35 °C for 7–10 days [45]. The separation of the biomass was carried out by centrifugation at 10,000 rpm for 10 min. The filtrate was then passed through 0.45 μm membrane filter (Merck, Darmstadt, Germany) separate bacterial cells from the culture medium.

### 4.5. Purification of the Antimicrobial Metabolite(s)

#### Extraction of the Antimicrobial Metabolite(s)

Extraction was carried out sequentially using solvent extraction method. The solvents used were ethyl acetate and dichloromethane. Ethyl acetate was added first to the filtrate in equal volumes in a separating funnel [45]. The mixture was agitated for 2 h at 10 min intervals and left-over night for complete separation. The upper organic layer was collected and stored 4 °C for further analysis. The previous steps were repeated for dichloromethane [45]. The organic layers from ethyl acetate and dichloromethane were dried using rotary evaporator (Buchi R205, Flawil, Switzerland) at 45 °C [44,46,47]. Extracts were examined for its antimicrobial activities using agar well diffusion technique and negative controls were prepared using dimethyl sulfoxide (DMSO).

### 4.6. In Vitro Testing of the Antimicrobial Activities of the Extracted Metabolite(s)

The crude extract from ethyl acetate and dichloromethane were both dissolved in DMSO [42]. The antimicrobial testing was performed using both extracts and the filtrate left after extraction with both solvents using well diffusion method [44]. The two organic extracts were tested against eight organisms (*S. aureus* ATCC 25293, VRSA2, KP1, KP2, EC1, EC2, *C. albicans* ATCC 10231, CA1). A negative a control well was filled with DMSO [44].

### 4.7. Characterization of the Antimicrobial Metabolite(s)

#### 4.7.1. Thin Layer Chromatography (TLC) Analysis

Preliminary separation of the metabolite(s) was carried out to the ethyl acetate extract using TLC analysis. The crude ethyl acetate extract was spotted on Silica TLC coated plates 20 × 20 cm (pre-coated with silica gel 60 F254, Merck, Germany) and developed in three different solvent systems. The three solvent systems were: ethyl acetate: methanol (9:1); Ethyl acetate: dichloromethane (9:1); and dichloromethane: methanol (6.5:3.5). The fractionated metabolites were observed under UV light at 365 nm (fluorescence) and 254 nm (absorbance) [48].

#### 4.7.2. Liquid Chromatography-Mass Spectroscopy (LC/MS) Analysis

LC/MS analysis was performed at Center for Drug Discovery Research and Development at Faculty of Pharmacy, Ain Shams University, Cairo, Egypt. The analysis was performed using ESI-MS positive and negative ion acquisition mode with a XEVO TQD triple quadruple instrument, Waters Corporation, Milford, MA01757 USA, mass spectrometer. The stationary phase was a ACQUITY UPLC—BEH C18 1.7 µm—2.1 mm × 50 mm Column (Santa Clara, CA USA). The mobile phase was gradient elution consisted of water containing 0.1% formic acid and acetonitrile containing 0.1% formic acid with flow rate of 0.2 mL/min.

### 4.8. Metagenomics Analysis of the Soil Samples

#### 4.8.1. DNA Extraction and Quantification

Metagenomics were carried out by HITS Solutions Co. (Bioinformatics Department, Cairo, Egypt, http://www.hitssolutions.com/, accessed on 7 November 2021). Qiagen DNeasy power-soil kit (Cat. no. 12888-50 Qiagen, Germany) was used for DNA extraction as per manufacturer protocol. After DNA extraction the DNA quantity was measured by Qubit fluorometer ver. 4.0 to ensure there is enough pure genomic material before the sequencing run. 400 ng/7 μL (55 ng/ μL), as mentioned by Oxford nanopore manual.

#### 4.8.2. Library Construction

Library construction was performed using Rapid Sequencing Kit (Oxford Nanopore Technologies, Oxford, UK, Cat. # SQK-RAD004). Before loading on the flow cell, a total of 34 μL of sequencing Buffer and 25.5 μL of loading Beads were added to 12 μL of the DNA libraries and 4.5 μL nuclease free water. After that priming and loading onto FLO-MIN106 flow cell were performed.

#### 4.8.3. Sequencing and Data Analysis

Sequencing was run on MinION™ (Oxford Nanopore Technologies, Oxford, UK) for 12 h which generates 3.03 M reads with N50 equals 9.29 K. Base calling was performed in real-time during sequencing by the Guppy software. which generates FAST5 and FASTq files, reads below Q7 were eliminated. Centrifuge was used to classify sequencing reads to a taxonomic identifier. A Centrifuge index was constructed using bacterial and viral genomes downloaded from NCBI RefSeq as of 03 March2017, and the human reference genome (GRCh38). Low complexity regions with a dust score greater than 20 in the reference sequences were masked using dust masker (v 1.0.0, NCBI). Results were visualized using re-centrifuged.

#### 4.8.4. Genome Sequencing Aligning and Analysis

From the previous step of soil sequencing analysis of the metagenome using antiSMASH version 2 (Antibiotics and Secondary Metabolite Analysis Shell) (http://antismash.secondarymetabolites.org/, accessed on 7 November 2021) for extraction probable secondary metabolite gene cluster of the isolate. Mauve software was used (http://gel.ahabs.wisc.edu/mauve, accessed on 7 November 2021) for draft genome comparison [17].

## 5. Conclusions

*A. faecalis* isolate MZ921504 is a promising source for antimicrobial metabolites with broad activities against MDR Gram positive and Gram-negative bacteria. The LC/MS analysis of *A. faecalis* metabolites confirmed the presence of ectoine, bacillibactin, quinolobactin and burkholderic acid. Metagenomics sequence analysis of the soil sample containing the respective isolate revealed the presence of conserved biosynthetic gene clusters of ectoine, bacteriocin, bacillibactin, quinolobactin, terpene and burkholderic acid. The obtained data confirmed the suitability of the metagenomic nanopore sequence analysis, which is a fast and reliable method for deciphering the nature of the active metabolites and potential is exhibited through cutting off the huge amount of time that was required for random antimicrobial discovery from natural sources.

## Figures and Tables

**Table 1:** Preliminary screening against Gram positive test organisms.

Isolate Code	Gram Positive Test Organisms					
	SE1	SE2	SE3	VRSA1	VRSA2	VRSA3
SS9	+	-	+	+	-	-
SS10	+	+	+	+	+	+
SS11	-	-	+	-	-	-
SS12	+	+	+	+	+	+
SS13	+	-	+	-	-	-
SS14	-	-	+	+	-	-
SS16	+	+	-	+	+	-
SS17	+	+	+	+	+	+

+: inhibits growth, -: no inhibition and SE1, *S. epidermidis* isolate 1; SE2, *S. epidermidis* isolate 2; SE3, *S. epidermidis* isolate 3; VRSA1, vancomycin resistance *S. aureus* isolate 1; VRSA2, vancomycin resistance *S. aureus* isolate 2; VSRA3, vancomycin resistance *S. aureus* isolate 3.

**Table 2:** Primary screening against Gram negative test organisms.

Isolate Code	Gram Negative Test Organisms					
	*E. coli* ATCC 25922	EC1	EC2	KP1	KP2	KP3
SS9	-	+	-	+	-	-
SS10	+	+	+	+	+	+
SS13	-	+	-	+	-	-
SS14	-	-	-	+	-	-
SS16	-	+	-	+	-	-
SS17	+	+	-	+	-	-

+: inhibits growth, -: no inhibition. EC1, MDR *E. coli* isolate 1; EC2; MDR *E. coli* isolate 2; KP1, MDR *K. pneumoniae* isolate 1; KP2, MDR *K. pneumoniae* isolate 2; KP3, MDR *K. pneumoniae* isolate 3.

**Table 3:** The antimicrobial activity of the of the solvent extracts of *A. faecalis* isolate MZ921504.

Test Organisms	Mean Zone of Inhibition (mm) ± SD	
	Dichloromethane Extract	Ethyl Acetate Extract
*S. aureus* ATCC 25293	13.0 ± 0.5	16.0 ± 0.5
VRSA2	-	16.0 ± 1.0
KP1	12.0 ± 0.5	17.0 ± 0.5
KP2	-	17.0 ± 0.5
EC1	15.0 ± 0.5	16.0 ± 1.0
EC2	-	13.0 ± 0.5
*C. albicans* ATCC 10231	-	17.0 ± 1.0
CA1	-	-

-, absence of inhibition zone. VRSA2, vancomycin resistance *S. aureus* isolate 2; EC1, MDR *E. coli* isolate 1; EC2; MDR *E. coli* isolate 2; KP1, MDR *K. pneumoniae* isolate 1; KP2, MDR *K. pneumoniae* isolate 2; CA1, *C. albicans* clinical isolate 1.

**Table 4:** Antimicrobial resistance profile of the bacterial clinical isolates.

Gram Positive		Gram Negative	
Clinical Isolate Code	Resistance Pattern	Clinical Isolate Code	Resistance Pattern
SE1, SE2, SE3	CLI, CN, FOX, CIP	KP1	AMC, ATM, CTX, CAZ, CRO, FEP, CIP, SXT, TET, IMP, ETP, DOR, CT, PB, FF, RA, CN
VRSA1	VAN, FOX	KP2	AK, AMC, ATM, CTX, CAZ, CRO, FEP, CIP, SXT, TET, IMP, ETP, DOR, CT, FF, RA, CN
VRSA2, VSRA3	VAN, CLI, CN, FOX, CIP	KP3	AK, AMC, ATM, CTX, CAZ, CRO, FEP, CIP, SXT, TET, IMP, ETP, DOR, FF, RA, CN
		EC1	CTX, IMP
		EC2	AK, AMC, ATM, CTX, CAZ, CRO, FEP, CIP, SXT, TET, IMP, ETP, DOR, FF, RA,

Glycopeptides: VAN = vancomycin, Macrolides: CLI = clindamycin Beta-lactams: AMC = Amoxicillin/clavulanic ATM = Aztreonam FOX = cefoxitin CTX = Cefotaxime CAZ = Ceftazidime CRO = Ceftriaxone FEP = Cefepime DOR = Doripenem ETP = Ertapenem IMP = Imipenem; Aminoglycoside: AK = Amikacin CN = Gentamicin Quinolones: CIP = Ciprofloxacin Polymyxins: CT = Colistin PB = Polymyxin B; Sulfonamides/Diaminopyrimidine: SXT = Sulfamethoxazole/Trimethoprim Tetracyclines: TE = Tetracycline; TGC = Tigecycline Phosphonic acid derivative: FF = Fosfomycin Rifamycins: RA = Rifamycin.

## Data Availability

Data are available within the article.

## Supplementary Materials

The following are available online at https://www.mdpi.com/article/10.3390/antibiotics10111382/s1, Figure S1: Metagenomics analysis of soil showing the diversity of *Alcaligens* species.
