# Supplementary material for: A Metagenomic Nanopore Sequence Analysis Combined with Conventional Screening and Spectroscopic Methods for Deciphering the Antimicrobial Metabolites Produced by Alcaligenes faecalis Soil Isolate MZ921504
Source: Antibiotics (Basel). 2021 Nov 11;10(11):1382. doi: 10.3390/antibiotics10111382 (PMC8614704; doi:10.3390/antibiotics10111382)
Supplement: Supplementary file 1 [file antibiotics-10-01382-s001.zip › antibiotics-1456137-supplementary.pdf]

Supplementary Material

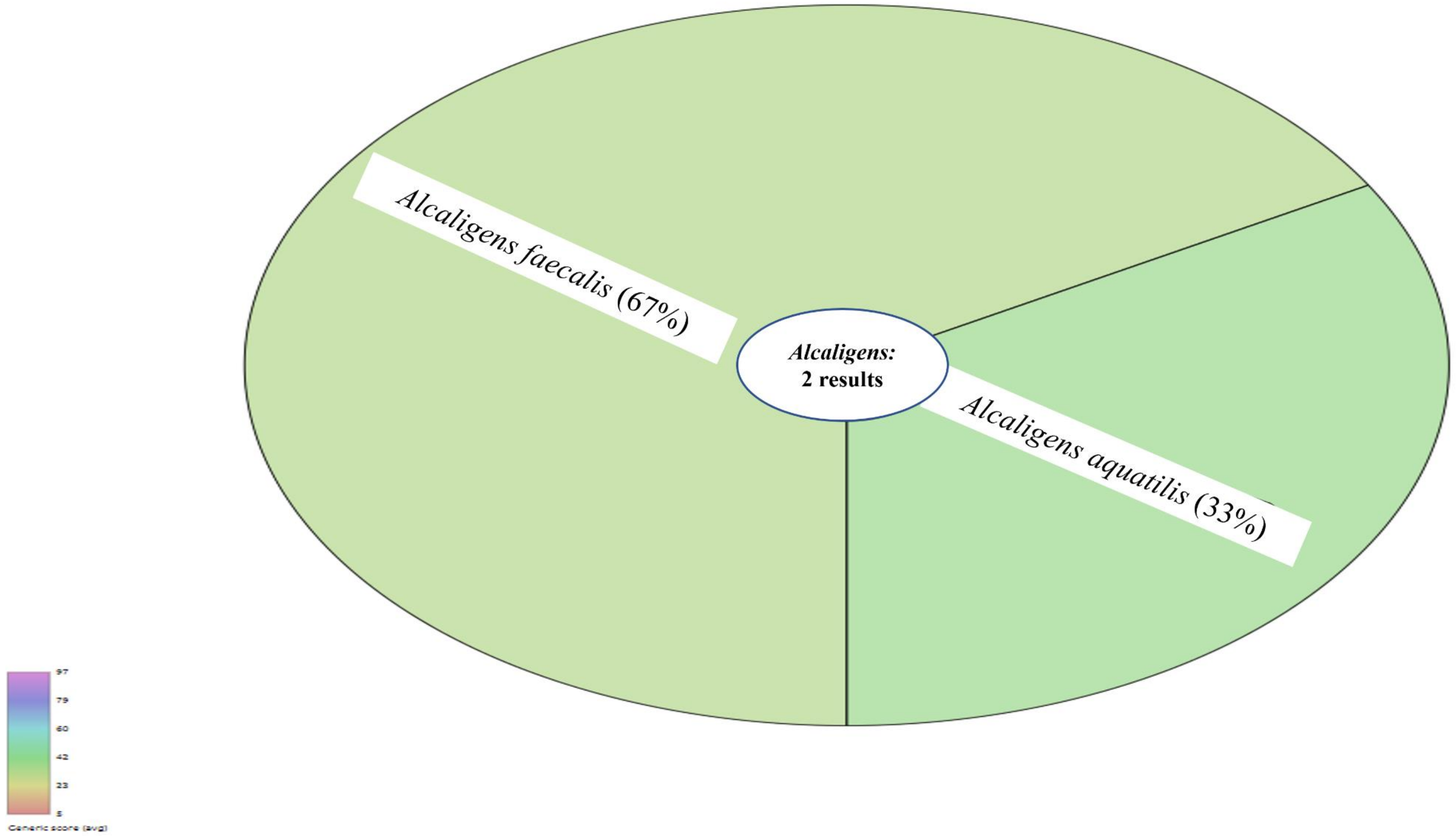

Figure S1. Metagenomics analysis of soil showing the diversity of *Alcaligens* species
